# Buschke-Löwenstein Tumor (BLT): Successful Treatment of Human Papillomavirus (HPV)-Associated Squamous Cell Carcinoma With Radical Surgery and Chemotherapy

**DOI:** 10.7759/cureus.72828

**Published:** 2024-11-01

**Authors:** Stanko J Baćo, Milos Mitric, Jovica Mišić, Igor A Stakic, Sonja Đukanović

**Affiliations:** 1 General Surgery, Public Health Institution Hospital “Dr Mladen Stojanović”, Prijedor, BIH; 2 General Surgery, Saint Luke the Apostle Hospital, Doboj, BIH; 3 Surgery, Private Hospital, Banja Luka, BIH; 4 Emergency Medicine, Public Health Institution Dom Zdravlja Prijedo, Prijedor, BIH

**Keywords:** buschke-löwenstein tumors, combined treatment, complex perianal fistulas, giant condyloma acuminatum, miles op, surgical excision

## Abstract

The giant condyloma acuminatum, known as the Buschke-Löwenstein tumor (BLT), is an uncommon, slow-growing, cauliflower-like tumor located in the anogenital region. It has a high recurrence rate, is sexually transmitted, and is often linked with immunosuppression. This tumor is commonly associated with human papillomavirus (HPV) infection, making HPV one of the most prevalent sexually transmitted infections affecting the perineal and genital regions. Treatment options primarily involve a combination of surgical excision and adjuvant chemotherapy. In this report, we present the case of a 63-year-old heterosexual, HIV-negative female patient whose locally advanced disease severely impacted her quality of life due to multiple bilateral perianal fistulas and the development of squamous cell carcinoma. The patient also struggled with chronic anemia resulting from recurrent hemorrhages. She underwent a Miles procedure, consisting of an extralevator abdominoperineal excision with terminal colostomy. Following adjuvant chemotherapy, she achieved complete wound healing by secondary intention, with no complications or signs of wart recurrence. This report highlights that surgical resection, complemented by adjuvant therapy, can lead to excellent outcomes even in advanced cases with local progression, regional spread, and malignant transformation, without requiring additional reconstructive surgery or skin grafting, thus facilitating recovery.

## Introduction

Buschke and Löwenstein first described giant condyloma acuminatum (GCA) as a penile lesion in 1925; however, it is now increasingly observed in the anorectal region, although malignant alterations are rare [1]. Despite its histologically benign nature, GCA exhibits clinical malignancy, frequently progressing to invasive squamous cell carcinoma (SCC). Although malignant transformation of condyloma acuminatum (CA) has been well documented on the skin and mucosal surfaces of male and female genitalia, reports of such transformation in the perianal area remain scarce [2].

Buschke-Löwenstein tumor (BLT) is defined by a prominent verrucous or cauliflower-like lesion predominantly affecting the anogenital regions. Histologically, these tumors demonstrate increased mitotic activity, papillomatosis, acanthosis, and a tendency to invade adjacent tissues [3]. Documented cases have also noted vertical transmission from mother to child. BLT is strongly associated with human papillomavirus (HPV) types 6 and 11, responsible for 90% of cases, with other subtypes occasionally implicated [3,4]. It is essential to rule out cutaneous angiosarcoma as a differential diagnosis, as it can resemble a large CA with molluscum-like satellite lesions [5].

BLT is thought to develop within a pathological spectrum, ranging from simple CA to SCC. An alternative view suggests that moderate CA lesions may often precede BLT formation. Its papillomatous appearance and size have led some researchers to classify BLT as an anogenital verrucous carcinoma, a well-differentiated variant of SCC [6]. In this article, we share our experience at a secondary hospital in a developing country, detailing the case of a 63-year-old female patient whose rapidly advancing disease led to multiple bilateral perianal fistulas and the onset of SCC in just six months.

## Case presentation

A 63-year-old female presented to our surgical unit with a complaint of a rapidly enlarging anal mass. The mass had reportedly been present for approximately six months and was associated with pruritus, pain, delayed defecation, persistent clothing soiling, and intermittent bleeding. Furthermore, the patient experienced severe chronic anemia due to repeated hemorrhages. She consistently took only antihypertensive medications. No information was available regarding her or her sexual partner’s high-risk sexual behavior, and she tested negative for HIV.

**Figure 1:**
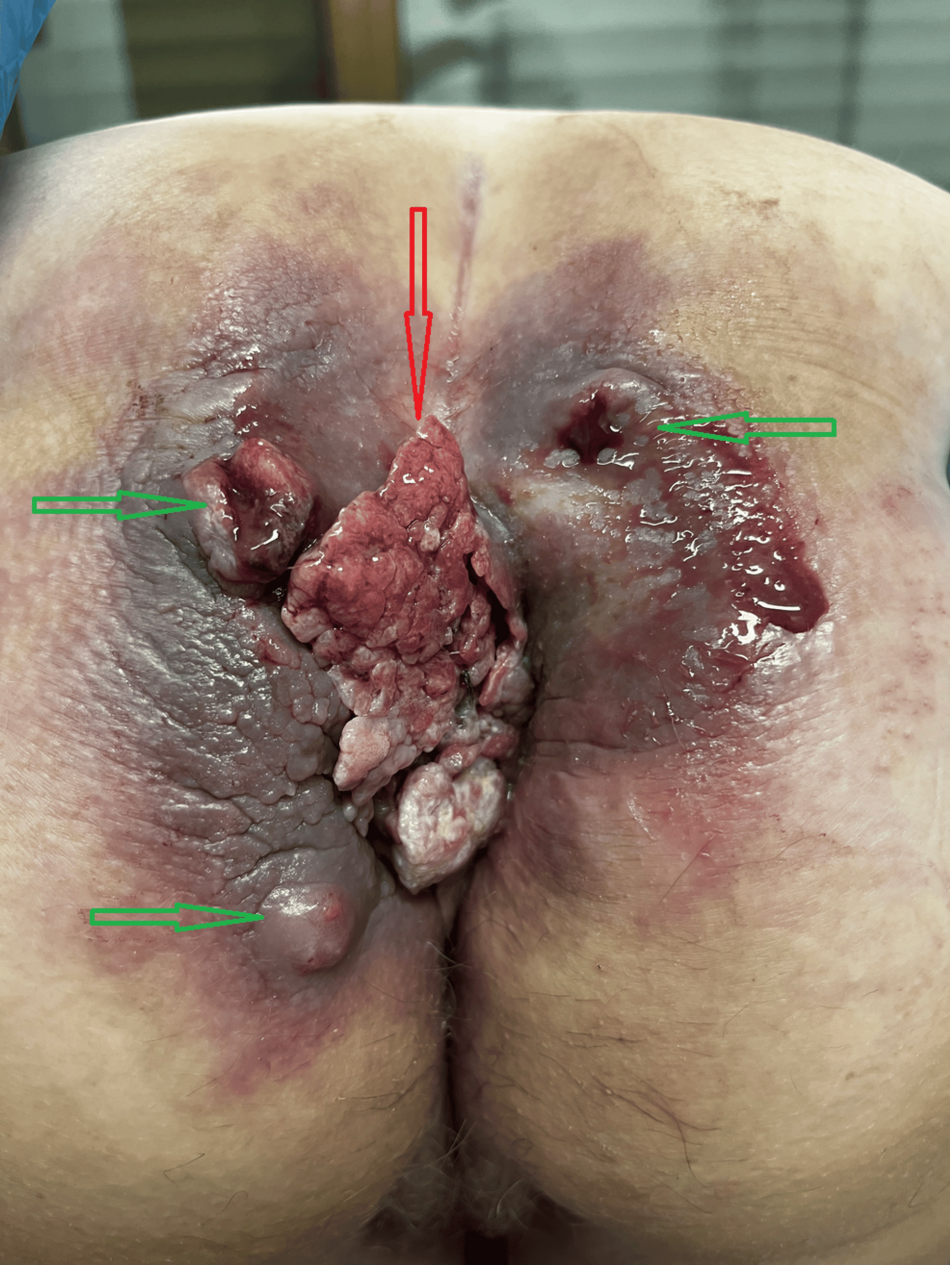
Initial examination of the perianal region in the knee-elbow position, showing a visible tumor (red arrow) and perianal fistulas (green arrows)

**Figure 2:**
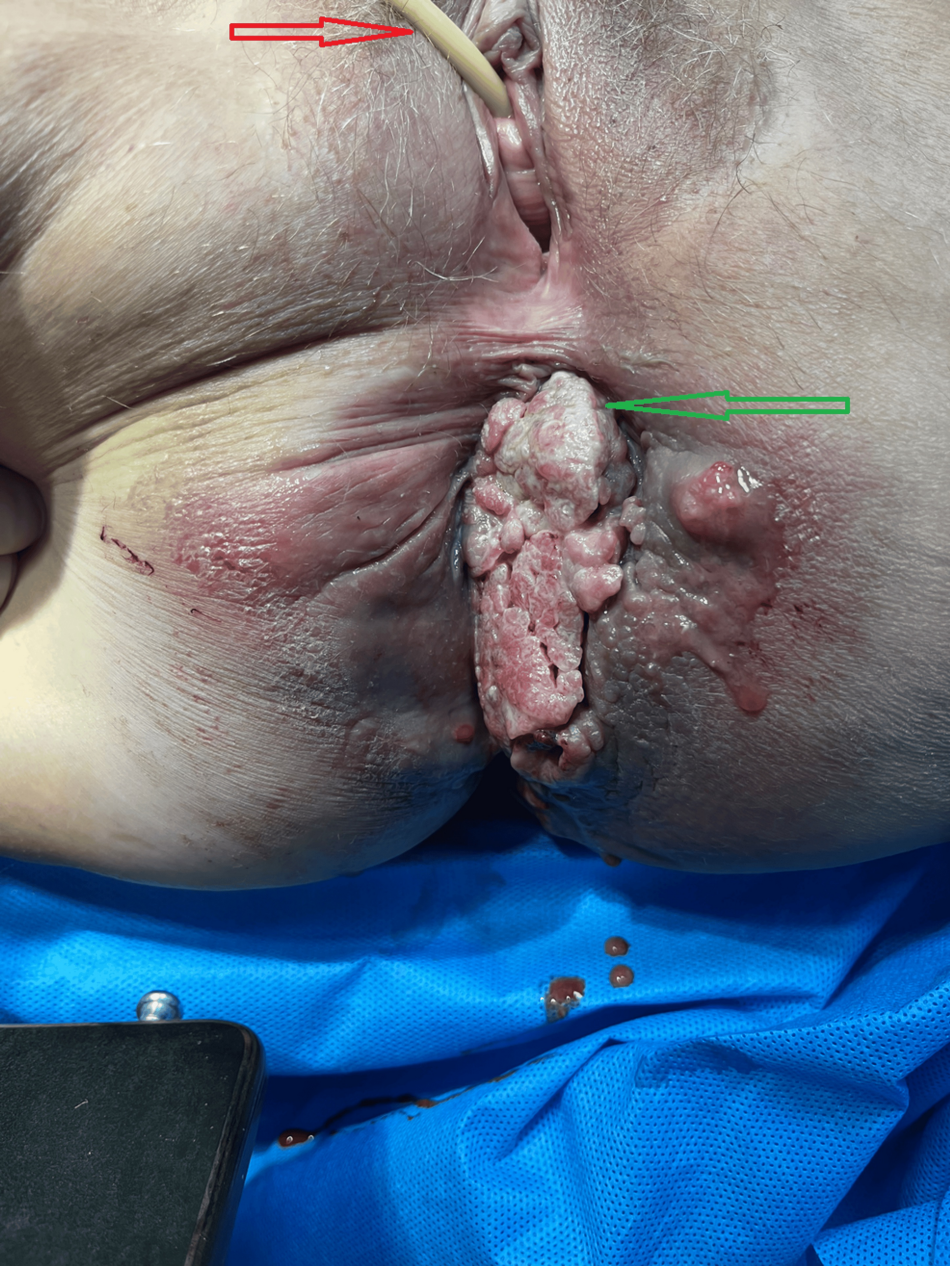
Preoperative view showing the tumor affecting the left perianal region (green arrow) and the urinary catheter placement (red arrow)

Upon examination, we identified a neoplastic growth on the left perimeter of the anal orifice. The mass resembled cauliflower, was fragile and susceptible to hemorrhaging, and had nearly occluded the anal aperture. Additionally, we observed several fistulas opening onto the perianal skin, accompanied by foul-smelling discharge (Figure 1, Figure 2). There was no enlargement of the inguinal lymph nodes.

**Figure 3:**
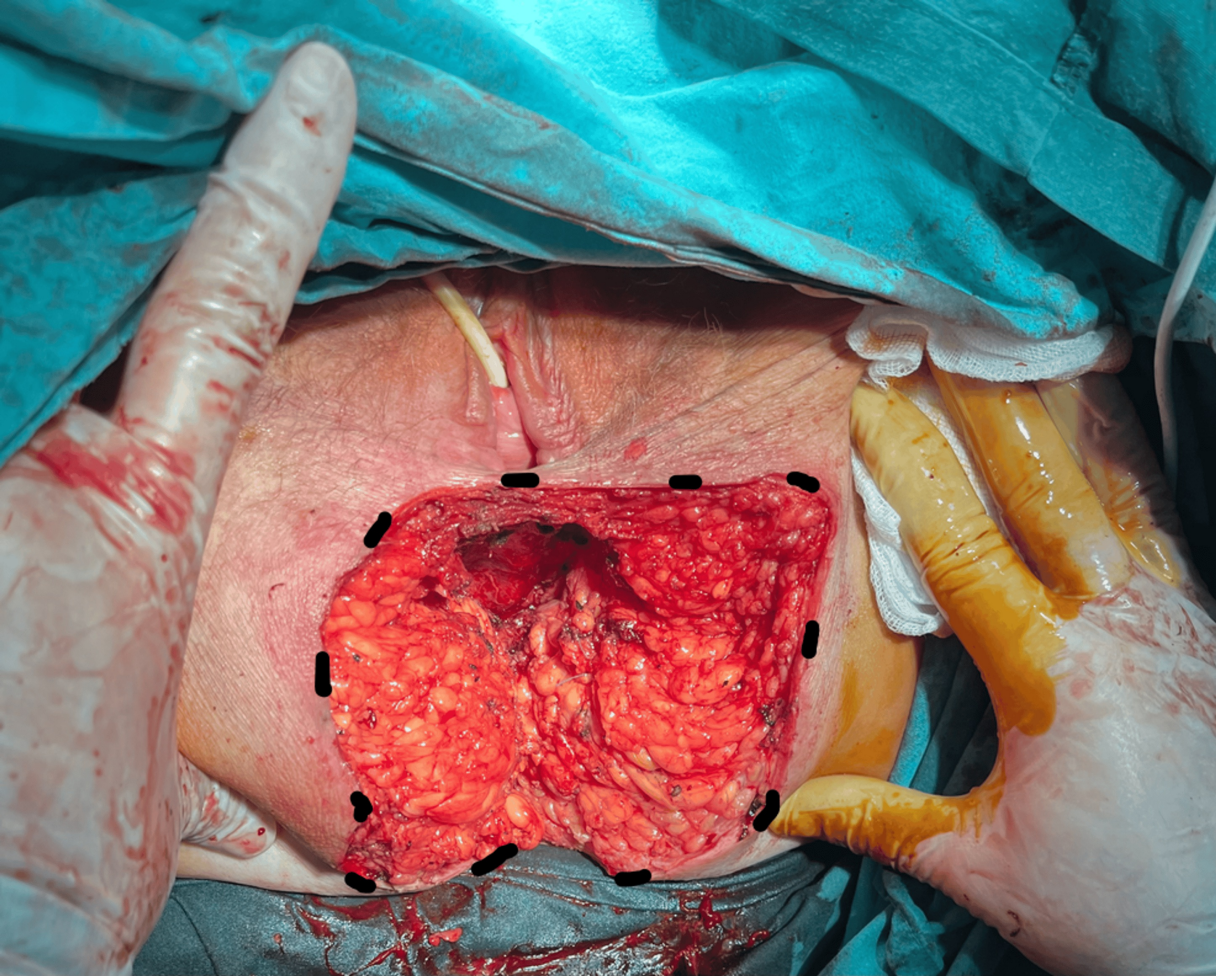
Immediate postoperative view showing the abdominoperineal wound, left to heal by secondary intention (outlined by black lines)

**Figure 4:**
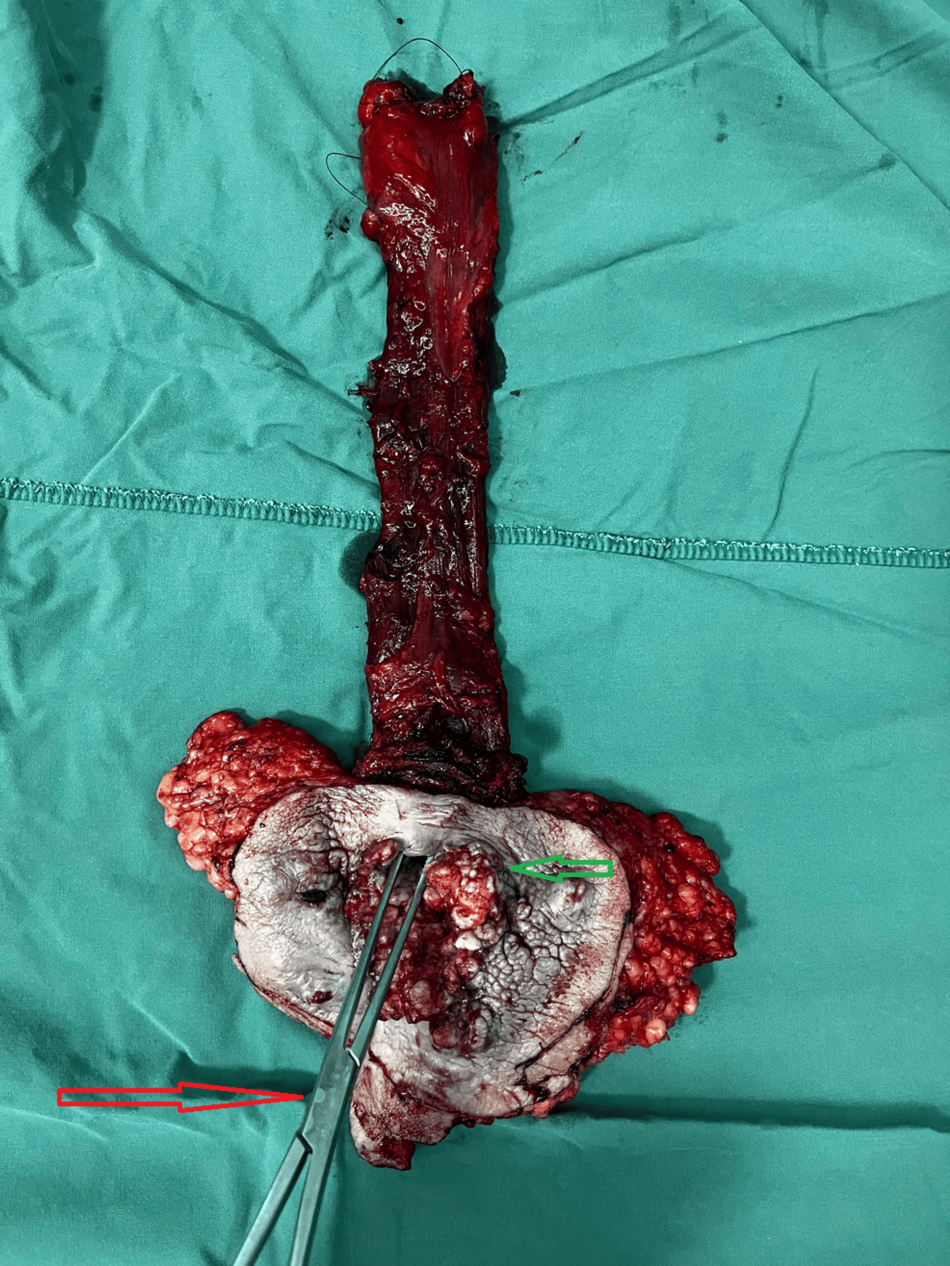
Excised specimen of the lower rectum showing neoplastic changes (green arrow) The instrument (red arrow) is positioned at the anal opening, which is notably close to the minimal opening.

Technical limitations and low image quality hindered the use of preoperative nuclear magnetic resonance (NMR) imaging from another facility as a guide for the extralevator abdominoperineal resection (Figure 3, Figure 4). The predetermined date of the operation, combined with the constraints of a resource-limited setting, precluded repeat NMR testing, and the patient insisted on proceeding with surgery during the same hospital admission.

**Figure 5:**
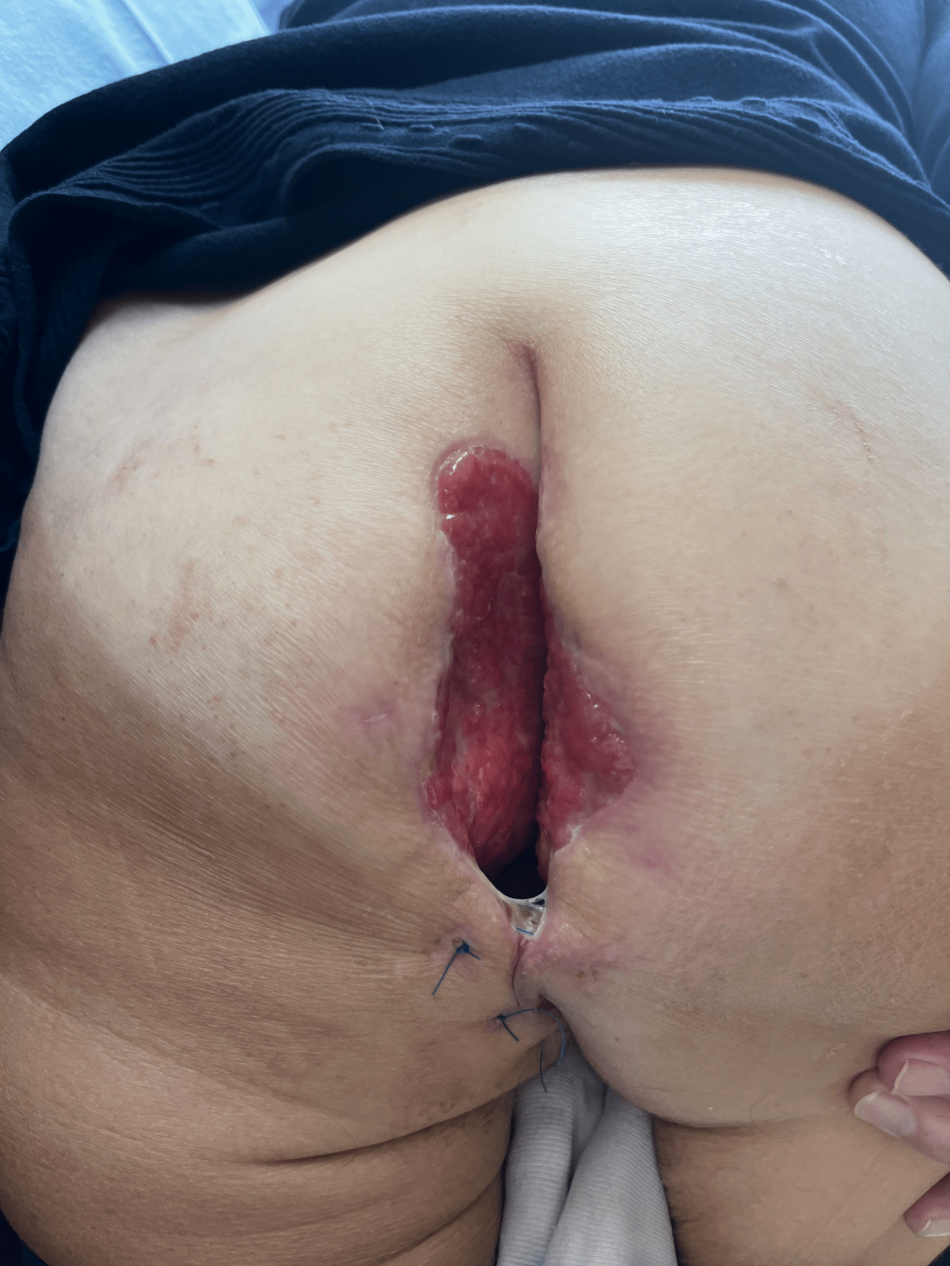
Local results 18 days post-surgery, showing a wound with clean, well-formed granulations

**Figure 6:**
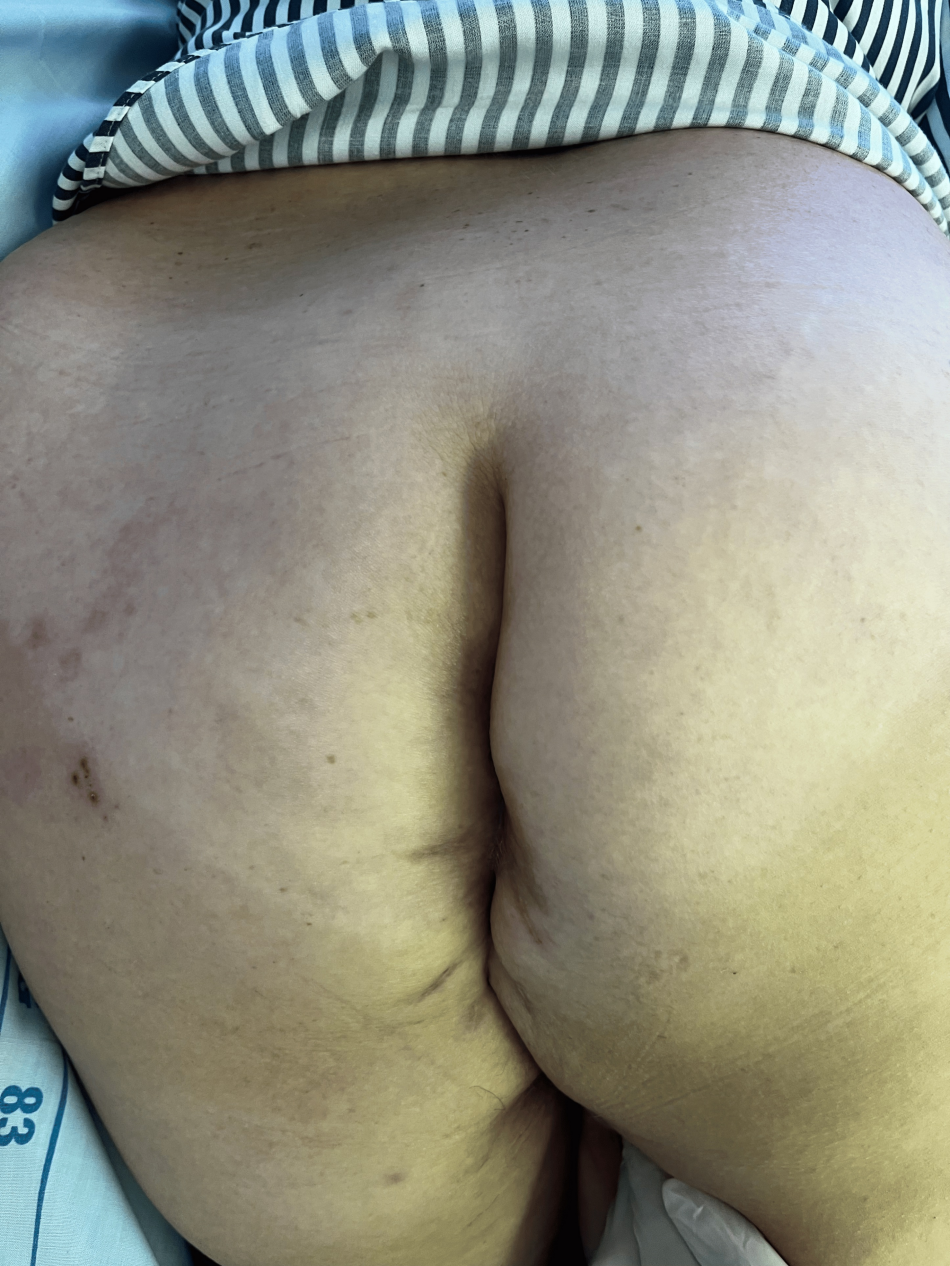
Condition six months after the completion of adjuvant chemotherapy and standard wound dressing

We performed the extralevator abdominoperineal resection with the patient in the prone jackknife position. The surgery lasted 110 minutes, and a terminal sigmoidostomy was created. The wound was left to heal by secondary intention (Figure 3, Figure 4, Figure 5, Figure 6).

The time for complete wound closure was four months, following a typical postoperative course that involved regular home wound dressings using only water and soap (Figure 5, Figure 6).

**Figure 7:**
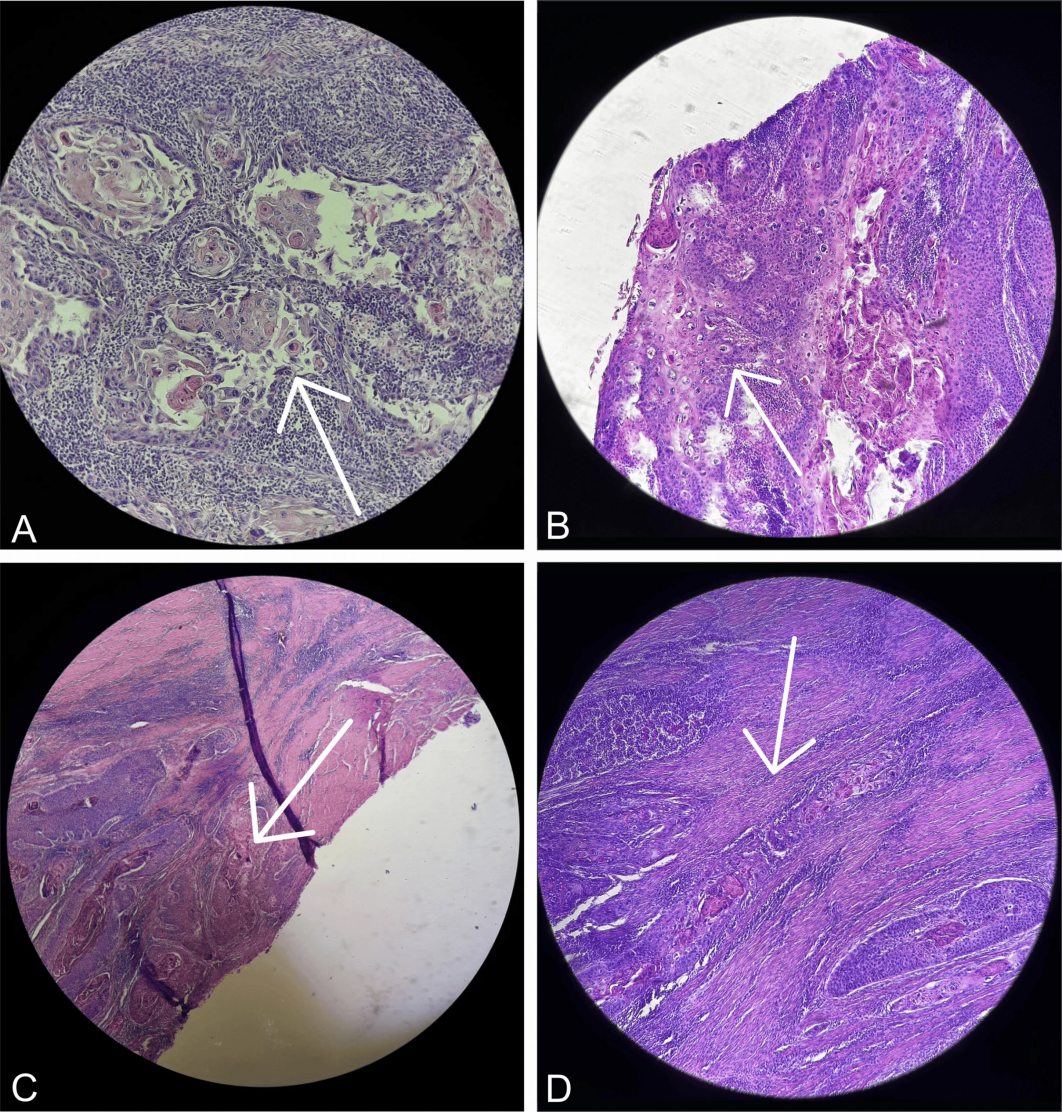
(A) Clusters of atypical squamous cells exhibiting disrupted nucleocytoplasmic ratios, enlarged irregular nuclei, coarse granular chromatin, excessive acidophilic cytoplasm, and keratinization. The stroma is densely inflamed. (B) The stroma contains irregular clusters of atypical squamous cells characterized by enlarged bizarre nuclei, coarsely granular chromatin, acidophilic cytoplasm with small keratinized centers, and pronounced koilocytic atypia. (C) Aggregations and patterns of atypical squamous cells with prominent centers and significant koilocytic atypia infiltrate the adjacent connective stroma. (D) Atypical squamous cells are arranged in irregular bands, with projections infiltrating the surrounding connective stroma..

**Figure 8:**
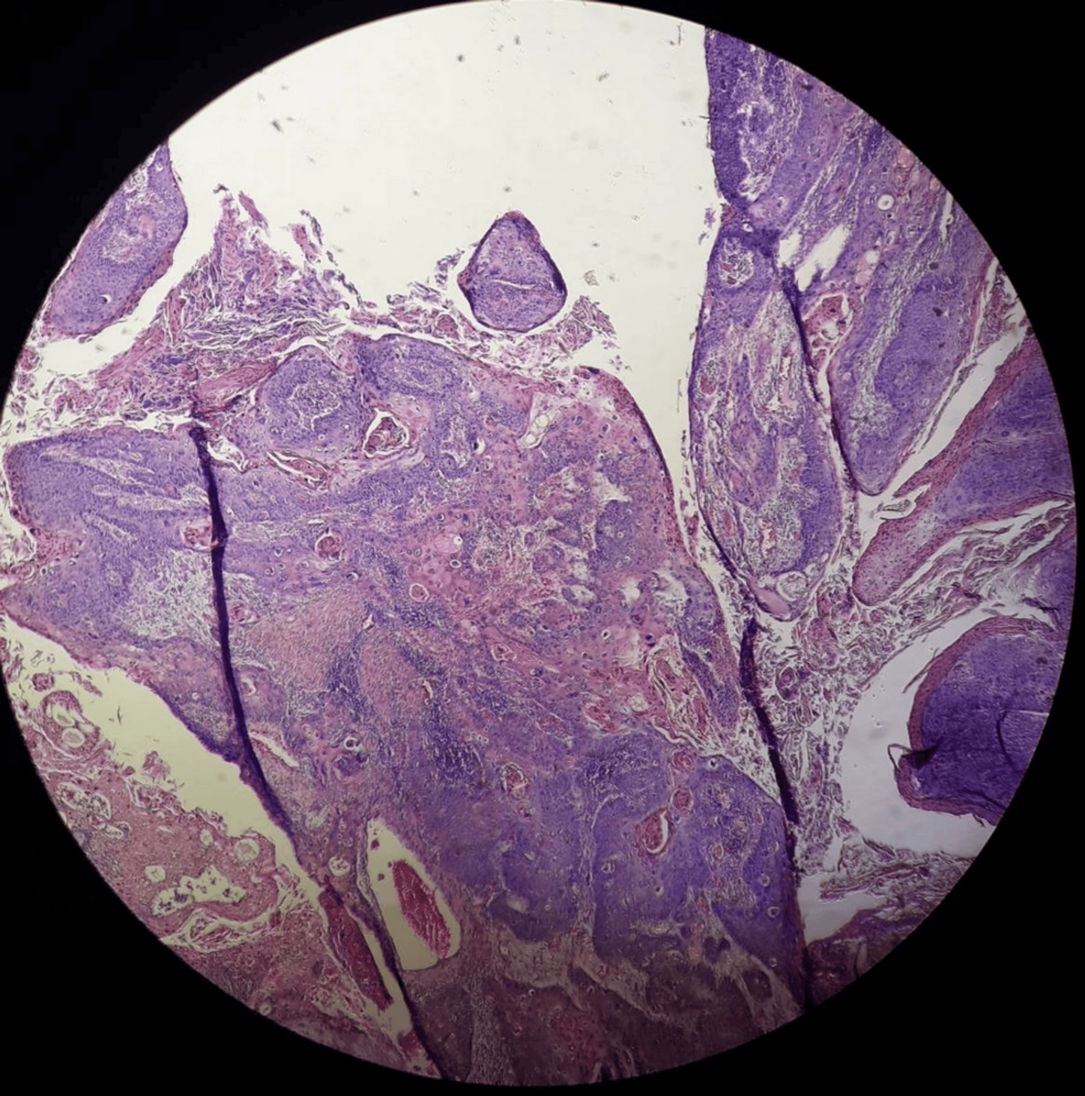
Atypical squamous cells exhibiting a disrupted nucleocytoplasmic ratio, characterized by oval to round enlarged nuclei and coarsely granular chromatin, line the villous outgrowths, which feature a central connective vascular core. The cytoplasm displays abundant acidophilia, and circularly distributed tumor cells coexist with keratinization.

Histopathologic examination (Figure 7, Figure 8) revealed that the Buschke-Lowenstein tumor had transformed into a Grade II SCC, classified as pT3NxMx, specifically associated with HPV. At an oncology conference, it was decided to administer four cycles of 5-fluorouracil combined with cis-diamminedichloroplatinum as adjuvant chemotherapy. A one-year follow-up demonstrated no recurrences and a significant improvement in the patient’s quality of life, with a completely healed wound and no restrictions.

## Discussion

This case highlights a rare occurrence of BLT in the anal region, characterized by destructive invasion and malignant transformation, which significantly compromised the patient’s quality of life due to its large size and associated complications. The context is further complicated by limited access to neoadjuvant therapy, with family members advocating for prompt intervention and results. This case underscores the importance of straightforward, uncomplicated, and safe surgical treatment for a locally advanced BLT that has undergone malignant transformation.

The GCA, commonly referred to as the BLT, is named after its initial proponents, Abraham Buschke, who first documented the lesion in 1896 in Neisser’s Sterokopischer Atlas [6], and Ludwig Löwenstein, who, together with Buschke, described another penile lesion in 1925 [6,7]. Dawson provided the first anorectal description of the disease in 1965 [8]. Serological data indicate that HPV infection is one of the most prevalent sexually transmitted diseases, affecting a significant portion of the population [9]. To date, nearly 450 HPV genotypes have been isolated and sequenced [10], with HPV genotypes 6 and 11 responsible for over 90% of condylomas, exhibiting moderate carcinogenic potential. The combination of genotypes 16 and 18, along with factors related to chronic irritation and decreased immunity, can lead to cancerous transformation and growth from both the exterior and interior.

Men aged 40-60 are most frequently affected by BLT, which typically arises in the genital area [11]. To prevent the progression and complications associated with CA, which is generally considered a benign condition, radical surgical intervention is recommended as soon as feasible [12]. There is currently no gold standard for treating this rare disease; thus, the experience and skills of the physician play a critical role in determining the treatment approach.

Nonsurgical treatments for GCA include topical therapies (such as podophyllin, fluorouracil, or radiation), tumor ablation methods (like cryotherapy with liquid nitrogen, CO2 laser therapy, electrocautery, or surgical excision), and immunotherapy (such as imiquimod), although their efficacy remains uncertain [13-18]. While podophyllin may benefit common acuminatum condylomas, it is ineffective for BLTs. Intralesional antivirals, like cidofovir, or topical immunomodulators, such as imiquimod cream (5%), may also be employed [15,16]. Following imiquimod treatment or surgical excision, carbon laser therapy may be utilized for large CAs [17]. Electrocoagulation with an electric scalpel is often applied for small condylomas; however, it increases the risk of recurrence. Depending on the extent of disease and proximity to critical structures, cytoreduction with immune-chemoradiotherapy may be considered pre- or postoperatively to reduce the need for mutilating surgery [17,18].

The persistence of numerous bilateral perianal fistulas, which severely impacted the patient’s quality of life, prompted initial considerations for radical surgical intervention. Following neoadjuvant radiochemotherapy, particularly radiotherapy alone, the pelvic area may experience the formation of new, difficult-to-heal fistulas, as well as anaplastic transformation and the extensive appearance of new condylomas [19]. This issue is compounded by the risk of anal stricture. Radiotherapy is contentious, as it appears to elevate the risk of anaplastic transformation and exacerbate preexisting condylomata acuminata. It is considered an optional treatment modality for managing giant perianal condylomata in selected cases [18,19].

The risk of recurrence, which can reach 60-70%, is closely tied to the necessity of achieving tumor-free margins. A major concern regarding surgical intervention is the increased likelihood of recurrence after both radical local excision and abdominoperineal resection; the latter is not recommended as the initial treatment unless significant pelvic or bone involvement is present [19]. The lack of sufficient patient series for the same technique and transient responses to topical, immunotherapy, and chemotherapy complicate the situation further [19]. Factors such as the presence of a fistula, involvement of the anal sphincters, or mucosal condition above the dentate line influence the degree of radical surgery required, suggesting that colostomy and abdominoperineal excision should be considered in specific cases [19].

Another significant concern is the tumor’s location and the presence of fecal matter, which could contaminate the surgical site and impede wound healing in the perianal region. Several studies recommend a temporary loop colostomy to mitigate the risk of fecal contamination, although this inevitably induces discomfort and limits patients' daily activities [19]. Treating BLT poses challenges, and even spontaneous remission may be unattainable; comprehensive surgical intervention remains the foundation of treatment, despite documented instances of rare self-resolution for BLT [20].

The limitations of this case report include restricted access to HPV testing and high-resolution NMR. The authors suggest that in cases of persistent, numerous bilateral perianal fistulas, aggressive surgical intervention may be warranted. One possible side effect of neoadjuvant radiochemotherapy is the development of new, difficult-to-heal fistulas in the pelvic area, which may affect nearby organs. The clinical implications of this are particularly significant in developing countries and regions where neoadjuvant therapy is not consistently available, making surgical treatment the primary approach.

## Conclusions

This paper examines a highly advanced, localized disease, with the objective of demonstrating that a comprehensive surgical approach, in conjunction with adjuvant chemotherapy, can yield significant results without necessitating additional reconstructive surgery that may hinder recovery. Diagnosis is confirmed through biopsy, pathohistological analysis, and further HPV testing. Immediate radical surgical intervention is essential to prevent the progression and complications associated with condyloma acuminata, despite its benign nature. Although rare, malignant transformation remains a critical concern, as does the potential for disease transmission to additional sexual partners.

Radical surgical intervention, specifically abdominoperineal resection following the Miles technique, can achieve excellent outcomes in cases of pelvic invasion or malignant transformation. This established and reliable method promotes healing of the perineal wound through secondary intention, particularly in the presence of complications and local advancement, as well as in resource-limited settings such as developing countries where access to neoadjuvant therapy is limited. The results of our surgical treatment approach are highly satisfactory. Additionally, we advocate for compulsory vaccination against the HPV virus.
